# Optimisation of simulated team training through the application of learning theories: a debate for a conceptual framework

**DOI:** 10.1186/1472-6920-14-69

**Published:** 2014-04-03

**Authors:** Martin Stocker, Margarita Burmester, Meredith Allen

**Affiliations:** 1 Neonatal and Paediatric Intensive Care Unit, Children’s Hospital Lucerne, Spitalstrasse, Lucerne 16 CH-6000, Switzerland; 2 Paediatric Intensive Care Unit, Royal Brompton Hospital, Sydney Street, London SW3 6NP, UK; 3 The Royal Children's Hospital, Department of Paediatrics, University of Melbourne, Victoria 3052, Australia

**Keywords:** Teamwork, Team training, In-situ simulation, Experiential learning theory, Socio-cultural learning theories, Conceptual framework

## Abstract

**Background:**

As a conceptual review, this paper will debate relevant learning theories to inform the development, design and delivery of an effective educational programme for simulated team training relevant to health professionals.

**Discussion:**

Kolb’s experiential learning theory is used as the main conceptual framework to define the sequence of activities. Dewey’s theory of reflective thought and action, Jarvis modification of Kolb’s learning cycle and Schön’s reflection-on-action serve as a model to design scenarios for optimal concrete experience and debriefing for challenging participants’ beliefs and habits. Bandura’s theory of self-efficacy and newer socio-cultural learning models outline that for efficient team training, it is mandatory to introduce the social-cultural context of a team.

**Summary:**

The ideal simulated team training programme needs a scenario for concrete experience, followed by a debriefing with a critical reflexive observation and abstract conceptualisation phase, and ending with a second scenario for active experimentation. Let them re-experiment to optimise the effect of a simulated training session. Challenge them to the edge: The scenario needs to challenge participants to generate failures and feelings of inadequacy to drive and motivate team members to critical reflect and learn. Not experience itself but the inadequacy and contradictions of habitual experience serve as basis for reflection. Facilitate critical reflection: Facilitators and group members must guide and motivate individual participants through the debriefing session, inciting and empowering learners to challenge their own beliefs and habits. To do this, learners need to feel psychological safe. Let the group talk and critical explore. Motivate with reality and context: Training with multidisciplinary team members, with different levels of expertise, acting in their usual environment (in-situ simulation) on physiological variables is mandatory to introduce cultural context and social conditions to the learning experience. Embedding in situ team training sessions into a teaching programme to enable repeated training and to assess regularly team performance is mandatory for a cultural change of sustained improvement of team performance and patient safety.

## Background

During the last decade medical and nursing authorities and societies have increasingly recognised the critical importance of team training as a mandatory domain for health professional education [1-4]. Suboptimal performance in non-technical skills (communication, leadership and teamwork) during critical events has repeatedly been shown to contribute to adverse events and poor patient outcomes [5-9]. The benefit of simulation training for non-technical skills for critical events has been shown in several studies to improve patient safety [10-16]. However, there is on-going debate as to which is the most effective way to provide simulation team training to health professionals [11-13,17-21].

Individuals bring assumptions about themselves, others and events to learning opportunities. These different views of reality are our personal “conceptual frameworks” [22]. A debate with critical appraisal of conceptual frameworks can lead educator and researcher to alternate views, with potential impact on design or assessment of educational programmes [22]. In a recent review of the literature regarding experimental studies in medical education, only half of the authors declared their conceptual frameworks [23]. We have recently published our simulated team training programme, with no reference to the underlying conceptual framework [24]. Debating the educational framework underlying a simulation programme may improve effectiveness, impact on team performance and hence patient safety. Hodges has recently advocated the use of bioscience, learning and sociocultural theories to design and conduct medical education programmes [25]. The following discussion will explore key educational frameworks and highlight aspects and debates that inform the development, design and delivery of an effective educational programme for simulated team training relevant to health professionals.

## Discussion

### Debate 1: Single versus repeated exposure in one training session

**Figure 1:**
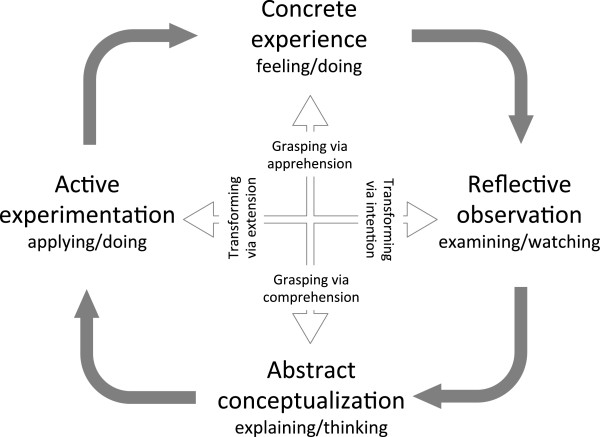
Kolb’s model of experiential learning.

What is the most effective way to structure a simulated team training session? Kolb’s learning cycle is currently the main conceptual framework used for experiential learning in simulation team training programmes [26-30]. Kolb defines experiential learning as a process by which knowledge is created through the transformation of experience [31]. In this model, true learning is depicted as a four-part process in a cycle (Figure 1). Individuals learn through concrete experience, reflection, conceptualisation, and experimentation. The cycle begins with the learner’s involvement in a specific experience (such as doing or feeling); then they reflect on the experience from a variety of perspectives (reflective observation such as examining or watching). Through reflection learners integrate their observations into more abstract models, create generalisations and principles and draw conclusions (abstract conceptualization such as explaining or thinking). The individual then uses these principles and conclusions to guide subsequent decisions and actions (active experimentation such as applying or doing) that lead to new concrete experiences [31].

**Figure 2:**
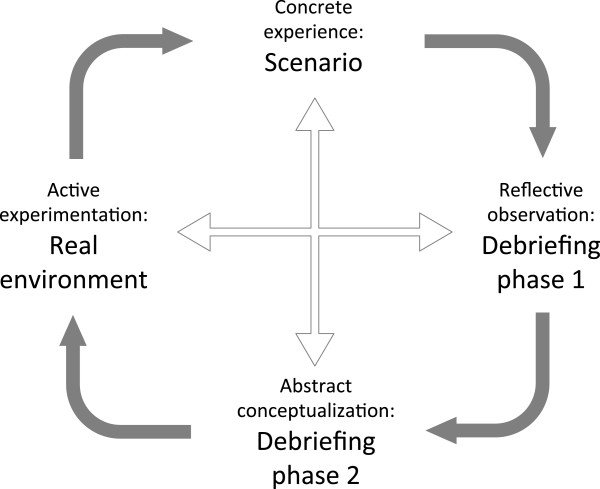
Simulated team training in the conceptual framework of Kolb’s experiential learning theory.

According to Kolb’s four-stage experiential learning cycle, immediate and concrete experiences are the basis for observations and reflections [31]. Consequently all participants in a simulated team training programme need to begin by completing a simulated critical event (Figure 2). They then come together for structured debriefing [26,29,32]. Debriefing addresses the second and third phases of Kolb’s cycle: reflective observation, then abstract conceptualization. Reflective observation describes observation and analysis of a concrete experience. This is mainly characterized through participants’ narrations and statements with reference to relevant experienced problems and situations that occurred during the simulated event. This is an emotional phase and comparable to brainstorming. Questions are asked and learners discuss different views and aspects of a problem [26,29,31,32]. According to Kolb, these reflections are then assimilated and distilled into abstract concepts from which new implications for action can be drawn [31]. These new implications have to be actively tested and serve as guides in creating new experiences. This is the fourth phase of Kolb’s cycle (active experimentation).

There is broad agreement regarding the first 3 phases of Kolb’s learning cycle in published frameworks and guidelines how to structure a simulated training session. However, the debate regarding structure is about the fourth phase (active experimentation). This part of the cycle is often not executed during the same simulation session if programmes finish following the debriefing by summarising the conclusions drawn and the principles of the abstract conceptualizations [12,16,18,24,26,29,30]. This means that active experimentation has to be completed later, on an individual basis in the clinical setting, or at another simulation session. According to Kolb, it is important for learners to go through all four steps for the learning to be effective [31]. There are three main outcomes due to omission of this active experimentation phase: First, on an individual basis it may not be possible for the learner to apply the concluded principles in a safe environment, without facing the risk of real adverse outcomes and reactions. Second, there is perhaps no feedback of the environment to the newly applied action. Both of these outcomes will discourage learners to apply a new, but not yet tested behaviour. Third, after a substantial time relapse, conceptualised but not tested changes may be lost. There is a real risk to return to actions based on habits and non-reflective experience. Thus, active experimentation through experiencing a second scenario after debriefing is preferable.

First Statement: An effective structure for a simulated team training session contains a scenario for concrete experience, a debriefing with a reflexive observation and abstract conceptualisation phase, followed by a second scenario for active experimentation. Let the learners go back in after the debriefing even if for only a part of the original scenario to try their new frames.

### Debate 2: Simple experience versus experience of failure

What do we know regarding the concrete experience as catalyst for effective learning? Kolb was not the only theorist on experiential learning. Knowles defined andragogy and summarised adult learning principles as follows: Learning is most effective if we can relate to previous experiences, if we are internal motivated and if it is relevant and problem-centred [33]. Therefore it is mandatory to build scenarios with relevant problems to the participants. Scenarios can be derived from real events to obtain well-staged, realistic scenarios with clinical relevance and optimal authenticity [24]. This recommendation is in line with published frameworks and guidelines regarding simulated team training [13,19,30].

The debate regarding experience is focused on the level of difficulty and the importance of failure. One of the corner stones of Kolb’s learning cycle is the concept of immediate individual experience as the basis of reflective observation. In contrast, Miettinen elaborates that Dewey’s theory of reflective thought and action regards not experience itself but the inadequacy and contradictions of the habitual experience as the basis for reflection [34]. This means, we are most motivated to reflect and learn if we feel inadequate. The need to solve problems arising from habitual actions drives reflective observation, conceptualization and experimental activity to test new principles and ways. Published reviews regarding simulation in healthcare education are less explicit regarding the level of difficulty, recommending that training should be across a wide range of difficulty, commencing at basic skills and proceeding progressively to higher levels of difficulty [13,19]. However, if failure is preferable in order to initiate and drive the learning process, training should be carefully targeted to the needs of the participants. Each simulated event must challenge team members by generating dissonance and failures in order to optimise efficiency of simulated team training and adult learning.

Second Statement: It is mandatory to challenge participants during simulated training to experience failures and difficulties that serve as starting point for reflective observations. Scenarios derived from real events, pitched to the learners’ background facilitate feelings of inadequacy that motivate to learn: The group must feel that they are operating at the edge of their comfort zone.

### Debate 3: Individual reflection versus critical reflection in the group

How should we reflect during debriefing in order to optimise learning? Jarvis modified Kolb’s learning cycle and developed a model with different possible ways taken in experiential learning situations [35]. Non-learning (learner does not respond to a specific learning situation), non-reflective learning (memorisation or acquisition of manual skills without necessity of reflexion) and reflective learning are possible end products. To minimise non-learning and non-reflective learning it is essential that during the debriefing session facilitators incite and empower learners to go through the process of reflective observation and abstract conceptualization. Conflict resolution between opposite principles and the integration of new, more precise or refined ideas, are a process of adaptation and creating knowledge. There is broad agreement regarding the necessity of feedback and guided reflection after the simulated experience [13,16,19,26,27,29,30].

This third debate questions the effectiveness of guided, individual critical reflection. In a recent published guideline regarding the use of reflection in medical education, Sandars elaborates educational strategies to develop reflection: i) Motivation; ii) development of metacognitive skills as noticing (through self-monitoring, feedback from others, and analysis of significant events), processing (reflection for learning, to develop a therapeutic relationship, and to develop professional practice), and informing future action; and iii) reflective storytelling and writing [27]. Schön assumes that individuals live in a world of insecurity, instability, complexity and conflict, where they often must deal with problems for which no existing rules or theories learned through formal training can apply [36]. Unexpected events, problems or surprises trigger two kinds of reflection. The first, “reflection-in-action”, occurs immediately by improvising an “on the spot experimentation”, thinking and testing out, refining and retesting various solutions for the problem. The second, “reflection-on-action” occurs when individuals reflect after the problem: They examine what they did, how they did it and what alternatives existed [36]. Some of us reflect in-action and share it with our teams at the time. All of us reflect-on-action but are usually not provided with a system to share/process/learn from this reflection. Schön says that critical reflection is more than simply reflecting-in or reflecting-on-action, one’s own conceptual framework must be questioned: why did I do what I did? What beliefs inform my practice and how are these beliefs helping or hindering my work [36]? Miettinen analysing Dewey’s theory of reflective thought and action regards individual observations as laden by prior conceptualisation and interpretation [34]. Learners need strong guidance of one’s peers and facilitators to truly reflect on self. It is highly unlikely that an individual would be able to observe unbiased experiences, reflect openly on these, conceptualise new ideas and principles, and apply these new concepts actively, without the pressure of inadequacy and facilitation through others [34]. Therefore it is important during debriefing that participants explore and discuss their experience in depth within their group. They need to discover which form of adaptation works best in a particular situation, and to challenge their own conceptual frameworks and principles. Participants need to feel secure in their group and motivated through their group members in order to challenge their own beliefs [27,34,37].

Third Statement: Facilitate critical reflexion. Debriefing is fundamental and there is a need for participants to challenge their existing frameworks and principles. To support critical reflection trained facilitators and peers are required to guide and motivate participants in a secure and open way: Let the group talk and critical explore.

### Debate 4: Improvised versus real teams

The motivation and preparedness of participants to challenge one’s own frameworks and principles may vary. Is it possible to enhance and activate this process within real teams? According to Bandura, people’s judgements of their own ability to deal with different situations (self-efficacy) is central to their actions [38]. He suggests that motivation and self-knowledge are two main areas that play an important role in self-efficacy, and that this is the major determinant of the goals a person will set, and of the energy, effort, and perseverance that will be dedicated to their achievement. Self-efficacy may or may not be accurate and arises from four main information sources: Performance attainment, observation of other people, verbal persuasion, and physiological state [38,39]. The focus of this debate is the impact of using real teams versus improvised teams on the learning process in simulated training.

According to Bandura, observing what can happen and drawing conclusions from experiences of others can also provide knowledge to the learner and influence self-efficacy [38]. Observation of others is not possible if Kolb’s learning cycle is taken on an individual basis. However, a team training programme allows the possibility of observing others during the concrete experience, followed by reflective observation and concrete conceptualisation. Verbal persuasion occurs through feedback of other participants as well as observing facilitators, during the reflective observation and concrete conceptualisation phases of debriefing. Learning is further enhanced when associated with a heightened physiological state (increased heart rate, sweating and muscle tension) from scenario engagement. In order to involve and challenge participants it is important to build realistic concrete experiences within real teams (i.e. high-fidelity mannequins, authentic scenarios obtained from real events, implementation of realistic care as possible). To correctly pitch a scenario enabling physiologically activated participants to derive insight into their response to stress for improvised teams is challenging due to previous unknown team expertise. In addition, interactions and feedback between team members with heightened physiological states may be much more pronounced within real teams than within improvised groups who may not know each other. Adopting Bandura’s principles to simulation team training programmes, it is therefore essential to involve real multidisciplinary teams with members of different specialities and levels of expertise. As a team it is possible for the individual learner to observe peers, gain insight into their own performance and to model behaviour and knowledge [39]. Several publications investigating the effect of resuscitation training demonstrated a positive enhancement of self-efficacy through personal performance mastery experience, observational learning, verbal persuasion and attention to the affective state of participants [40-42].

Fourth Statement: Real team members of different specialities and levels of expertise support motivation and preparedness of participants for effective learning: Make the gap between simulation and reality as small as possible.

### Debate 5: Simulation centre versus in-situ simulation

Most studies reporting simulated team training are done in the setting of a simulation centre. Recently published guidelines and reviews regarding simulation based training request a safe environment and an implementation into a curriculum without specific discussion regarding the debate simulation setting [6,7,10,12-14,19]. The classical learning theories and Kolb’s model have recently been criticized by Bleakley et al. because they refer to the individual learner and not the team or system, and so critical elements of learning are missed [43,44]. Bleakley advocates the use of socio-cultural learning models in order to provide a more powerful tool for understanding how learning occurs in complex, dynamic systems such as teams. The assumption that knowledge, mind and memory are not just individual, but distributed across persons and artefacts, is one of the key points of socio-cultural models: Knowledge is permanently negotiated by members of the team and common knowledge is more than the sum of individual recollections [43]. In a socio-cultural approach learners are not at the activity centre, they are just one aspect in a complex system where learning is sensitive to the context, and gaining access to activity is crucial.

The most prominent theory expanding learning from acquisition to participation in dynamic social contexts is the activity theory of Engeström [45]. An activity system describes multiple actions of different people with a shared goal (the object). Each activity system should be considered as a whole and goal-directed actions are always explicit or implicit, characterised by ambiguity, surprises, interpretations, sense making, and potential for change. The system is influenced and mediated by the social setting, and participation necessarily acts as a disturbance to an already unstable system that offers productive possibilities through change over time [45]. According to this model, a simulated team training session can be thought of as an activity system. Key questions during the session are then about the interplay of the different individuals, each with their different history, role, dispositions and concerns. No individual mind is essential, but the distributed cognitive system with shared knowledge and skills. Significant changes in the system result not from individual decisions but from critical shifts in states of the system (team responding to a crisis). To use a simulated event as an activity system there is a need for real multidisciplinary teams with members of different expertise, acting in a standard set-up environment, with authentic and realistic events with clinical relevance. In this case, learning can be described as a system-based activity and the basic unit of analysis is a functional team operating through time. Socio-cultural learning theories therefore indicate that in-situ team training simulation sessions improve effectiveness and efficacy of learning in preference to training in a simulation centre.

Fifth Statement: It is mandatory to include the social and cultural context of a team for effective team training. Real teams acting in their standard environment (in-situ simulation) is the key to introduce context to the programme.

### Conceptual framework

**Figure 3:**
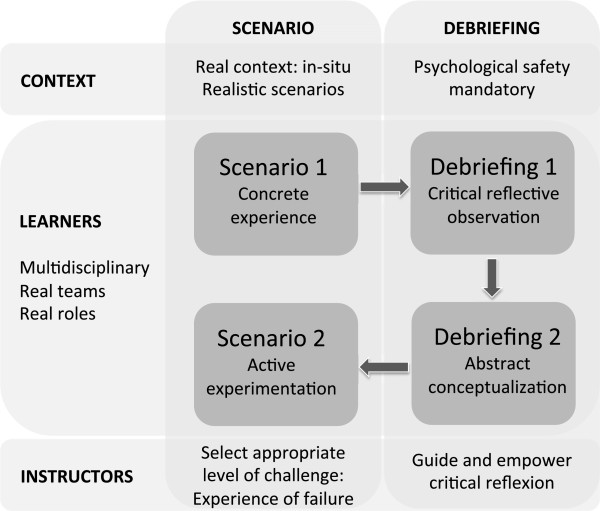
Conceptual framework for effective simulated team training.

We have applied different models of experiential learning theories, constructivism and of sociocultural theories to create a conceptual framework for the design and delivery of an optimal simulated team training programme (Figure 3). Our aim has been to outline different theories illuminating different aspects of learning in simulated team training and to combine these aspects to a concise and feasible framework (scholarship of integration and application). There are also several recently published guidelines and frameworks regarding learning through simulation using different strategies [4,12,13,18,19,21,30,46],[47]. In order to investigate our work it is necessary to compare our new conceptual framework with these published frameworks.

Kneebone based his framework on his experience, observations and on different learning theories [47]. He used the model of Ericsson regarding deliberate practice to emphasise gaining technical proficiency, Vygotsky’s “zone of proximal development” to illustrate the place of expert assistance, and Lave and Wenger to highlight the importance of learning within a professional context. Kneebone proposed 4 criteria to critically evaluate simulation training: Simulation should i) allow for sustained, deliberate practice within a safe environment; ii) provide access to expert tutors; iii) map onto real life clinical experience; and iv) provide a supportive, motivational, and learner-centred milieu. All these required principles are mapped to a certain extent in our framework. Deliberate praxis is essential for sustained change. This agrees with our first statement regarding the structure of the simulated team training. We focused on the structure of one training session requesting a second scenario to allow participants to experiment new applied frames. For sustained improvement of team performance simulated team training should be embedded in a programme thus enabling regular, repeated sessions [4,13,19,21,24]. The request for access to expert tutors maps to our third statement: expert facilitators select an appropriate level of difficulty during the scenario and guide and empower participants to critically reflect during the debriefing phase. This is in line with Kneebone’s criteria to provide a supportive, motivational and learner-centred milieu, a message supported as well by simulation studies outside of the area of team training [48]. Our second and fifth statements encompass the criteria to map onto real life clinical experience, indeed we request more specifically to create experiences at the edge of the participants’ comfort zone and within the clinical environment (in-situ simulation).

Zigmont used adult and experiential learning theories to propose a framework for developing and facilitating simulation courses [30]. For effective practice based learning he focused on 3 areas: The individual (previous knowledge, self efficacy and psychological safety), the learning environment and key experiences. Within these 3 areas he elaborated important principles as for example key experiences have to be challenging, emotionally charged and contains mistakes and errors. These principles are mapped to our second, third and fourth statements. Feedback culture and a culture to change are important factors of the environment area of Zigmont’s framework [30]. We agree that these factors are mandatory for sustained change within a clinical environment. In-situ simulation with real teams (4^th^ and 5^th^ statements) may offer the possibility fostering organizational learning and culture change [49].

Berragan published a framework conceptualising learning through simulation based on sociocultural theories [46]. Benner and Sutphen’s concept of situated knowledge in practice and Engeström’s activity theory are used to explore learning during simulation. The focus of this framework is the formation of professional identity, contextualisation of care and development of professional competency. This framework based on sociocultural learning theories is in line with our 4^th^ and 5^th^ statements. Our framework, in addition, investigates the transfer of learning theories into practice as well as the principles of experiential learning and aspects on motivation and reflection.

Cheng recently published a review for instructors regarding simulation-based crisis resource management (CRM) [18]. His guidelines are broadly based CRM-principles with description and appraisal of scenario design, debriefing strategies and assessment tools. The described debriefing strategies are very similar supporting our first and third statements. The focus of assessment of teamwork during simulated team training is an aspect not covered in our framework. This is an omission due to our approach applying theories that are focused on learning and not assessing. Assessment is important for feedback and remediation [50] and depends upon the content of the training session (i.e. CRM principles, technical skills) and available resources. Assessment tools for simulation training are not yet sufficiently validated or focused on teamwork [51]. Clearly, assessment is mandatory for a teaching programme and regular, longitudinal assessments may be a suitable approach with impact on learning and patient safety [52,53].

Two recently published reviews regarding simulation in healthcare education broadly support our first, second and third statements [13,19]. Curriculum integration, deliberate practice and assessment are aspects not sufficiently covered in our framework due to our focus on the single simulation session. Undoubtedly, it is mandatory to embed simulated team training into a teaching programme in order to enable repeated training and to therefore foster optimal teamwork and patient safety [24]. In-situ simulation is a newer strategy with the advantage to be within the usual context and working environment. This provides an opportunity to address organisational and system-based processes within the original cultural and social context [21]. In our framework, emphasizing the sociocultural context (fourth and fifth statements) in simulated team training is probably the most significant difference to other published frameworks, and this may lead to better learning outcomes.

There are several limitations of our proposed framework as developed through the application of learning theories. The main limitation is the dependency on learning theories without application and validation of the proposed framework. Reliability and validity of the framework have to be evaluated and the impact on learning needs to be compared to other published guidelines. Second, our framework is focused on a single simulation session. Published literature shows the necessity of embedding simulated team training into a curriculum with the possibility of repeated training and deliberate practice. Third, assessment of team performance is mandatory for feedback and remediation and this aspect is not covered within our framework.

The strengths of our debate are the comparison and discussion of diverse learning theories, their application to simulated team training, the outcome of several statements describing the important aspects of a training session and the conclusion with a concise and feasible framework.

## Summary

There are several implications following the debate and critical appraisal of relevant learning theories as a conceptual framework for simulated team training programmes. There is always a gap between simulation and real clinical life. Simulation-based education can complement, but should not replace education involving real patients in genuine settings. Nevertheless, simulation team training can serve as a powerful tool and environment for learning. To be fully effective it is important to critically appraise the programme, to explicitly acknowledge and name the conceptual frameworks used and to compare them with known learning principles.

Statement 1: Kolb’s experiential learning theory prescribes mandatory steps for effective simulated team training sessions: Scenario for concrete experience, followed by a debriefing with a critical, reflexive observation and abstract conceptualisation phase, and ending with a second scenario for active experimentation. Let the learners go back in after the debriefing even if for only a part of the original scenario to try their new frames. The debate is regarding the second scenario. Omission of the second experimentation phase means no possibility to apply new frames in a safe environment, no guaranteed feedback of new applied actions and after substantial time relapse risk of losing conceptualised but not tested behaviours. Let them re-experiment to optimise the effect of a simulated team training session.

Statement 2: Other experiential learning theorists inform us that the scenario needs to challenge participants to generate failures and feelings of inadequacy to drive and motivate team members to critical reflect and learn. The debate is regarding the importance of failure during the experience. The inadequacy and contradictions of the habitual experience (rather than the experience itself) serve as a basis for reflection. A scenario generating dissonance and difficulties optimises the efficiency of simulated team training.

Statement 3: Debriefing is fundamental to reflection on action and Schön’s theory is that there is a need for participants to challenge their existing frameworks and principles. Facilitators and peers must guide and motivate participants through the debriefing session, inciting and empowering critical reflexion. To do this, learners need to feel psychological safe. The debate is regarding the effectiveness of individual, critical reflexion. Individual observations are laden by prior conceptualisation and interpretation and it is highly unlikely that an individual learner is able to observe unbiased experience, reflect critically challenging habitual frameworks and conceptualise new principles. Use the impact of all group members to drive and motivate individual participants to challenge their own beliefs.

Statement 4: Bandura’s theory of self-efficacy proposes that real multidisciplinary team members acting within their speciality and roles support motivation and preparedness of participants for effective learning. The debate is regarding the impact of real compared to improvised teams. Interactions between team members to heighten physiological state, to observe peers gaining insight into their own performance and to model behaviour may be much more pronounced within real teams compared to improvised teams not knowing each other. Use real teams fostering and supporting preparedness and motivation to improve their own team performance.

Statement 5: Socio-cultural learning theory proposes that it is mandatory to introduce cultural context and social conditions to the learning experience for effective team training. The debate is regarding team training in a simulation centre versus in-situ simulation. Knowledge is permanently negotiated by members of a team and learners are just one aspect in a complex system where learning is sensitive to the context. The system is influenced and mediated by the social setting and the context. Significant changes result not from individual decisions but from the team shifting in critical states during their response to a crisis. Use in-situ simulation to introduce the social and context setting into the training to improve effectiveness and efficacy of the learning session.

We created a conceptual framework applying the 5 statements coming out of different learning theories. We compared our new framework with other published frameworks and guidelines regarding simulated training. All statements are to some extent included in recently published guidelines and different frameworks, whereas there is no publication referring to all 5 statements. In contrast to others, our proposed framework emphasises the social setting and context together with the request for real multidisciplinary teams emphasising in-situ simulation for optimal team training. Curriculum integration, deliberate practice and assessment of team performance are aspects of other publications regarding simulated training not covered in our framework. Embedding in-situ team training sessions into a teaching programme in order to enable repeated training and to assess regularly team performance is mandatory for a sustained improvement of team performance and patient safety.

## Competing interests

We declare that we have no conflicts of interest.

## Authors’ contributions

All authors fulfil the standards for authorship. MS was responsible for the concept, drafted the manuscript and had the final responsibility of the published version. MB and AM made substantial contributions to the concept, were involved revising the manuscript critically and gave final approval of the published version.

## Authors’ information

MS works as consultant at the neonatal and paediatric intensive care unit at the children’s Hospital of Lucerne. He is director of the interdisciplinary simulated team and resuscitation training programme (iSTaRT) at his institution. MB has the clinical lead at the paediatric intensive care unit at the Royal Brompton Hospital in London, UK. She is director of the simulated paediatric resuscitation team training programme (SPRinT) at her institution. MA is director of medical education and clinical associate professor at the Royal Children’s Hospital, University of Melbourne, Australia.

## Pre-publication history

The pre-publication history for this paper can be accessed here:

http://www.biomedcentral.com/1472-6920/14/69/prepub
